# Evaluating the effects of temperature and agitation on biofilm formation of bacterial pathogens isolated from raw cow milk

**DOI:** 10.1186/s12866-024-03403-4

**Published:** 2024-07-08

**Authors:** Meshari Alabdullatif

**Affiliations:** Department of Pathology, College of Medicine, Imam Mohammad Ibn Saud Islamic University (IMSIU), Uthman Ibn Affan Rd, Riyadh, 13317-4233 Saudi Arabia

**Keywords:** Biofilm formation, Raw cow milk, Bacterial pathogens, Temperature, Agitation

## Abstract

**Objectives:**

To study the effect of agitation and temperature on biofilm formation (cell aggregates embedded within a self-produced matrix) by pathogenic bacteria isolated from Raw cow milk (RCM).

**Methods:**

A 40 RCM samples were gathered from eight dairy farms in Riyadh, Saudi Arabia. After bacterial culturing and isolation, gram staining was performed, and all pathogenic, identified using standard criteria established by Food Standards Australia New Zealand (FSANZ), and non-pathogenic bacteria were identified using VITEK-2 and biochemical assays. To evaluate the effects of temperature and agitation on biofilm formation, isolated pathogenic bacteria were incubated for 24 h under the following conditions: 4 °C with no agitation (0 rpm), 15 °C with no agitation, 30 °C with no agitation, 30 °C with 60 rpm agitation, and 30 °C with 120 rpm agitation. Then, biofilms were measured using a crystal violet assay.

**Results:**

Of the eight farm sites, three exhibited non-pathogenic bacterial contamination in their raw milk samples. Of the total of 40 raw milk samples, 15/40 (37.5%; from five farms) were contaminated with pathogenic bacteria. Overall, 346 bacteria were isolated from the 40 samples, with 329/346 (95.1%) considered as non-pathogenic and 17/346 (4.9%) as pathogenic. Most of the isolated pathogenic bacteria exhibited a significant (*p* < 0.01) increase in biofilm formation when grown at 30 °C compared to 4 °C and when grown with 120 rpm agitation compared to 0 rpm.

**Conclusion:**

Herein, we highlight the practices of consumers in terms of transporting and storing (temperature and agitation) can significantly impact on the growth of pathogens and biofilm formation in RCM.

## Introduction

Raw milk refers to milk that unpasteurized or otherwise treated to kill harmful bacteria [1]. Several studies have shown that consumers are motivated to drink raw milk by its perceived superior taste, nutritional value, and health benefits [2, 3]. In addition, lactic acid bacteria, which are commonly found in raw milk, have been shown to have antimicrobial activity against pathogens [4]; however, raw milk consumption is well established to be associated with high risk of foodborne disease [5].

Pathogenic bacteria, such as *Staphylococcus aureus*, *Streptococcus agalactiae*, *Klebsiella pneumoniae*, *Escherichia coli*, *Listeria monocytogenes*, and *Salmonella typhimurium* have been identified as a cause of food poisoning outbreaks linked to raw milk in several countries [6–8]. These bacteria can be present in the milk from the start or be introduced at any point during production and processing [9]. Public health strategies focus on reducing the risk of harmful bacteria in raw milk throughout the food supply chain, before it reaches consumers [10, 11]. Though, several studies have shown that consumer food handling can counterbalance food safety practices during processing and culminate in foodborne disease [12]. In particular, poor consumer food handling practices, such as leaving refrigerated foods like milk out at room temperature for too long, can create conditions that favor bacterial growth [13].

During milk storage and processing, the capacity of bacteria to form clumps of cells encased in a matrix of their own making, called biofilms, might contribute to their missed detection during routine screening [14–16]. Importantly, several studies have reported that bacterial biofilm formation can be increased at high temperature (~ 30 °C) [17, 18]; however, unfortunately few studies have focused on the effect of vehicle movement (agitation) on biofilm formation in raw milk [19, 20].

In this study, we collected raw cow milk (RCM) from eight dairy farms with the aim of isolating pathogenic bacteria. We then evaluated the impact of temperature (4 °C, 15 °C, 30 °C) and agitation (0 rpm, 60 rpm, and 120 rpm) on biofilm formation by the isolated bacteria.

## Materials and methods

### Sample collection

Raw milk samples were taken from storage tanks (300 to 3000 L in volume) at eight dairy farms in Riyadh, Saudi Arabi. A total of 40 samples were collected: one from each of five different bulk tanks at each farm, with approximately 30 mL per sample. Sample temperature during collection and the distance from the farm to the laboratory recorded. The samples were collected and transported to Imam Mohammad Ibn Saud Islamic University within two hours, where they were kept cold and tested the same day [19]. This study did not involve any human or animal subjects, so ethical approval was not required.

### Detection of presumptive pathogenic isolates

To identify bacteria in RCM, 6 mL of each sample (out of 30 mL total) was used as follows: First, 3 mL was streaked on six plates (0.5 mL per plate) using 5% sheep blood agar (SBA; Watin Biolife, KSA) for the identification of bacterial species with low loads. Second, the remaining 3 mL was diluted 10 times in phosphate-buffered saline (PBS) with a pH of 7.4, and the diluted sample was then spread on SBA for the determination of bacterial species with high loads. Plates were kept for 48 h at 37 °C under aerobic and 5% CO_2_ conditions.

Colonies were isolated for differentiation of pathogenic and non-pathogenic bacteria from the initial cultures based on several factors including: colony morphology (size, shape, color, and texture), diameter, hemolytic properties, and basic chemistry tests including catalase and coagulase assays. Furthermore, following isolation, gram staining (BD, NJ, USA) was performed as recommended by the stain manufacturer to differentiate between Gram-positive and Gram-negative bacteria.

Isolated colonies were incubated another two days at 37 °C under aerobic or in the presence of 5% CO_2_. Two colonies were picked and used to start a culture in 3 mL of sterile sodium chloride solution (0.45%), which was made to be as cloudy as a 0.5 McFarland standard. The bacterial suspensions were then put on each of two testing cards, VITEK 2 ID-GPB and VITEK 2 ID-GNB, and placed into a VITEK 2 system (bioMérieux) following the instructions from the manufacturer for species identification. In the event the VITEK 2 identification was made with less than 90% confidence. To ensure that the tests were being performed correctly, the laboratory also tested the following strains of bacteria, as recommended by the Clinical and Laboratory Standards Institute (CLSI): *Enterococcus faecalis* ATCC 29,212, *Streptococcus equi* subsp. zooepidemicus ATCC 43,079, *Escherichia coli* ATCC 35,218, *Pseudomonas aeruginosa* ATCC 27,853, *Staphylococcus aureus* ATCC 29,213, and *Klebsiella pneumoniae* ATCC 700,603 [19, 21].

### MALDI-TOF MS identification

We confirmed the identity of isolated bacteria by using MALDI-TOF MS system (bioMérieux) [22]. We followed the manufacturer’s instructions for the VITEK MS system to perform this analysis. Briefly, a loop was used to pick individual bacterial colonies and transfer them to designated spots on a slide. A special solution (VITEK MS-CHCA matrix) was then applied and allowed to dry completely. The prepared slide was loaded into the VITEK MS system. This instrument analyzed the bacterial proteins in each sample, generating a unique fingerprint. Since these fingerprints are primarily based on ribosomal proteins, they can effectively differentiate between different bacterial species. The generated fingerprints were compared to a database containing known fingerprints of various bacteria. The closer the match between a sample’s fingerprint and a database entry, the higher the confidence score assigned by the instrument. *Enterococcus faecalis* ATCC 29,212, *Streptococcus equi* subsp. zooepidemicus ATCC 43,079, *Escherichia coli* ATCC 35,218, *Pseudomonas aeruginosa* ATCC 27,853, *Staphylococcus aureus* ATCC 29,213, and *Klebsiella pneumoniae* ATCC 700,603 were used as the quality control strain.

### Resolving discrepancies with gene sequencing

Discrepancies between VITEK2 and MALDI-TOF MS were resolved by 16s rRNA sequencing as described by Geo et al. [23], which performed at King Faisal Specialist Hospital & Research Centre (Riyadh, Saudi Arabia). The results would be considered valid only if the similarity (homologous rate) between the sequenced gene and a known reference gene was above 99%.

### Biofilm formation

By using crystal violet assay to measure biofilms, pathogenic bacteria that had been identified were streaked on plates of trypticase soy agar (TSA; Watin Biolife) and grown at 37 °C for a night. A single colony was picked and used to start a culture in 3 mL of trypticase soy broth (TSB). The culture was incubated at 37 °C with shaking at 250 rpm for 24 h. The overnight cultures were then added to five wells of a 96-well plate (Corning Inc. Corning, NY, USA) in triplicate and grown to a final concentration of ~ 10^7^ CFU/mL in 180 µL (final volume) of TSB supplemented with 0.5% glucose (TSBg; Difco). To evaluate the effects of temperature and agitation on biofilm formation, the plates were then incubated for one day under the following conditions: first plate, 4 °C with no agitation (0 rpm); second plate, 15 °C with no agitation; third plate, 30 °C with no agitation; fourth plate, 30 °C with 60 rpm agitation; and fifth plate, 30 °C with 120 rpm agitation [24, 25]. We selected 30 °C as the temperature for agitation testing to reflect the average temperature in Riyadh, Saudi Arabia. After biofilm formation, gently washing was conducted on each well three times to remove planktonic cells with phosphate-buffered saline (PBS; pH7.4), and the biofilms were stained with 180 µL of 0.3% crystal violet (BD, MD, USA) for 30 min. The wells were then washed three times with PBS to remove any excess stain. Next, the wells were incubated with 180 µL of destaining solution (20% acetone and 80% ethanol) for 30 min to dissolve the bound stain. Samples of the stained biofilms were then transferred to a new plate and the absorbance was measured at 492 nm in a microplate reader (Apollo LB913, Bad Wildbad, Germany). The absorbance of the negative controls (TSBg alone with no bacteria added) was subtracted from the absorbance of the bacterial cultures [24–26]. As a control *Staphylococcus aureus* ATCC 29,213 (weak biofilm former) and *Klebsiella pneumoniae* ATCC 700,603 (strong biofilm former), were used in this study.

### Statistical analysis

Each experiment was repeated three times, with three replicates per repetition. The mean and standard deviation (SDs) of the measurements were calculated using Excel software. A mixed-model analysis was performed using SAS software (SAS institute, Inc., version 9.4) to adjust for multiple comparisons, and differences between groups were considered statistically significant if the *p*-value was less than 0.01.

## Results

### Prevalence of bacterial contamination in RCM

Of the eight farm sites, three exhibited non-pathogenic bacterial contamination in their raw milk samples. Of the total of 40 raw milk samples, 15/40 (37.5%; from five farms) were contaminated with pathogenic bacteria. Overall, 346 bacteria were isolated from the 40 samples, with 329/346 (95.1%) considered as non-pathogenic and 17/346 (4.9%) as pathogenic. The 17 isolated pathogenic bacteria were (number): *S. aureus* (4), *E. coli* (4), *K. pneumoniae* (2), *L. monocytogenes* (3), *S. agalactiae* (1), and *S. typhimurium* (3). Interestingly, only one farm (site C) had two pathogens detected in the same sample, which occurred twice: *S. aureus* and *E. coli* in sample number 6, and *S. agalactiae* and *S. typhimurium* in sample number 8 (Table 1).

**Table 1:** Pathogenic bacteria isolated from farm sites, temperature during collection, and distance between collection site and laboratory

Farm site	Contaminated milk sample	Pathogenic isolated bacteria	Temperature (^o^C) of collected sample	Approximate total distance (km) between farm to laboratory
A	1	*S. aureus* 20,101	4.9	45
	2	*S. aureus* 20,104	4.2	
	3	*E. coli* 20,102	13.7	
B	4	*K. pneumoniae* 20,103	26.9	83
	5	*E. coli* 20,105	15.4	
C	6	*S. aureus* 20,202 and *E. coli 20,203*	24.2	139
	7	*L. monocytogenes* 20,214	17.9	
	8	*S. agalactiae* 20,206 *and* *S. typhimurium* 20,207	25.8	
D	9	*E. coli* 20,204	22.4	91
	10	*L. monocytogenes* 20,205	16.9	
	11	*K. pneumoniae 20,208*	26.1	
E	12	*S. typhimurium* 20,210	30.2	120
	13	*L. monocytogenes 20,211*	26.6	
	14	*S. aureus* 20,212	27.4	
	15	*S. typhimurium* 20,213	29.9	

Regarding raw milk storage temperature, ten instances of contamination by pathogenic bacteria were found in milk stored above room temperature (more than 24 °C), five in milk at medium temperature (10 °C to 24 °C), and two in milk at low temperature (less than 10 °C). It is noteworthy that both instances of low-temperature contamination occurred at one farm (site A) and involved the same bacterial species, *S. aureus* (Table 1).

Regarding the relationship of transport distance to pathogenic bacterial contamination of milk, three instances of contamination (two with *S. aureus* and one *E. coli*) occurred in samples from farms at less than 30 min driving distance (< 50 km), five instances (two *E. coli*, two *K. pneumoniae*, and one *L. monocytogenes*) from farms 30 min to one hour away (50 km to 100 km), and nine instances (two *S. aureus*, one *E. coli*, two *L. monocytogenes*, one *S. agalactiae*, and three *S. typhimurium*) from farms more than one hour away (> 100 km). All told, our data support that higher storage temperature and longer transport distance increase the chance of isolating a pathogenic bacterium from raw milk samples.

*Matched results by the two identification methods* Among the 15 isolated pathogens tested, VITEK-2 and MALDI-TOF MS demonstrated a high level of agreement (93%). Fourteen isolates yielded the same identification at the species level using both methods. However, a single isolate (7%) showed a discrepancy. VITEK-2 identified it as *S. agalactiae* (*S. agalactiae* 20,206), which was confirmed by gene sequencing for added confidence. Intriguingly, MALDI-TOF MS identified this same isolate as *Nocardia asteroids*.

### Effect of temperature on biofilm formation

**Fig. 1:**
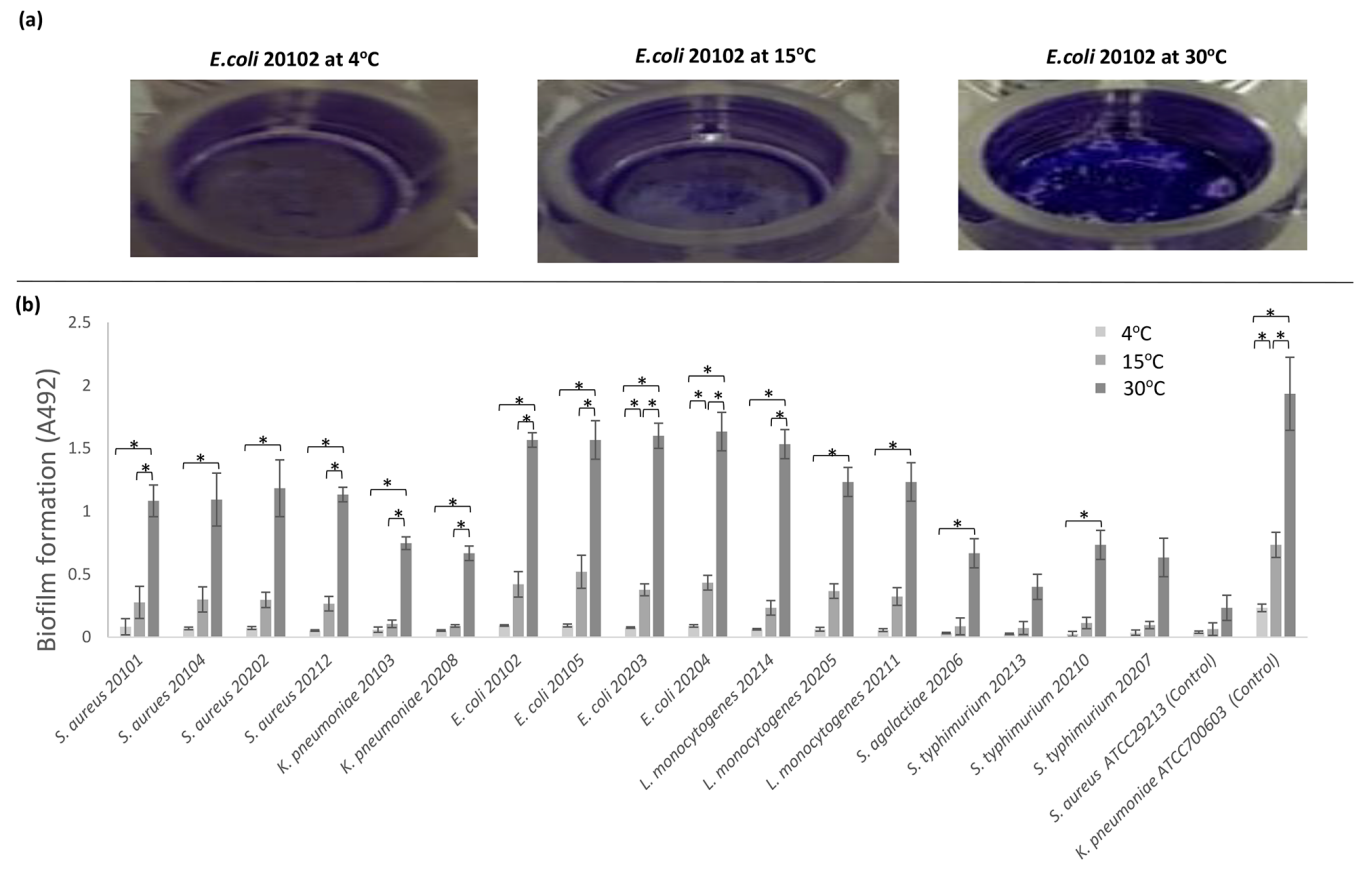
Effect of temperature on biofilm formation of isolated pathogenic bacteria in TSBg. Biofilm formation of E. coli strain 20,102 cultured in TSBg under different temperatures (4, 15, and 30 °C) was analyzed in a semi-quantitative manner using crystal violet assays **(a)**. Biofilm production was assessed as the optical density of stained biofilms at 492 nm. Data are presented as mean ± SD of three separate experiments with three replicates per experiment. Asterisks (*) indicate a significant (*p* < 0.01) difference in bacterial biofilm formation **(b)**

Different types of pathogenic bacteria isolated from raw milk stuck to plastic plates more or less strongly depending on the temperature at which they were grown (Fig. 1). According to Hassan et al., the ability of a bacterium to form a biofilm can be classified as weak, moderate, or strong [27]. When cultured at 4 °C, all isolated bacteria displayed weak biofilm formation, with OD_492_ values of 0.02 ± 0.02 to 0.09 ± 0.01. The pathogen with the highest biofilm formation at 4 °C was *E. coli* 20,102, and that with the lowest was *S. typhimurium* 20,210. At 15 °C, all isolated bacteria displayed weak to moderate biofilm formation, with OD_492_ values of 0.07 ± 0.05 to 0.52 ± 0.13. The pathogen with the highest biofilm formation was *E. coli* 20,105, and that with the lowest was *S. typhimurium* 20,213. Finally, when grown at 30 °C, all isolated bacteria displayed strong biofilms, with OD_492_ values of 0.40 ± 0.05 to 1.63 ± 0.13. The pathogen with the highest biofilm formation was *E. coli* 20,204, and that with the lowest was *S. typhimurium* 20,213 (Fig. 1b).

In general, all isolated pathogenic bacteria exhibited a significant (*p* < 0.01) increase in biofilm formation when grown at 30 °C compared to 4 °C; the exceptions were *S. typhimurium* 20,213 (*p* = 0.023) and *S. typhimurium* 20,207 (*p* = 0.026). When comparing 30 °C to 15 °C, *S. aureus* 20,101, *S. aureus* 20,212, *L. monocytogenes* 20,214, all *E. coli* strains, and all *K. pneumoniae* strains showed a significant (*p* < 0.01) increase in biofilm formation; other strains showed a trend towards increase, with *p* values between 0.046 and 0.018. Surprisingly, when comparing growth at 15 °C against 4 °C, only *E. coli* 20,203 and *E. coli* 20,204 exhibited a significant (*p* < 0.01) increase in biofilm formation (Fig. 1b).

### Effect of agitation on biofilm formation

**Fig. 2:**
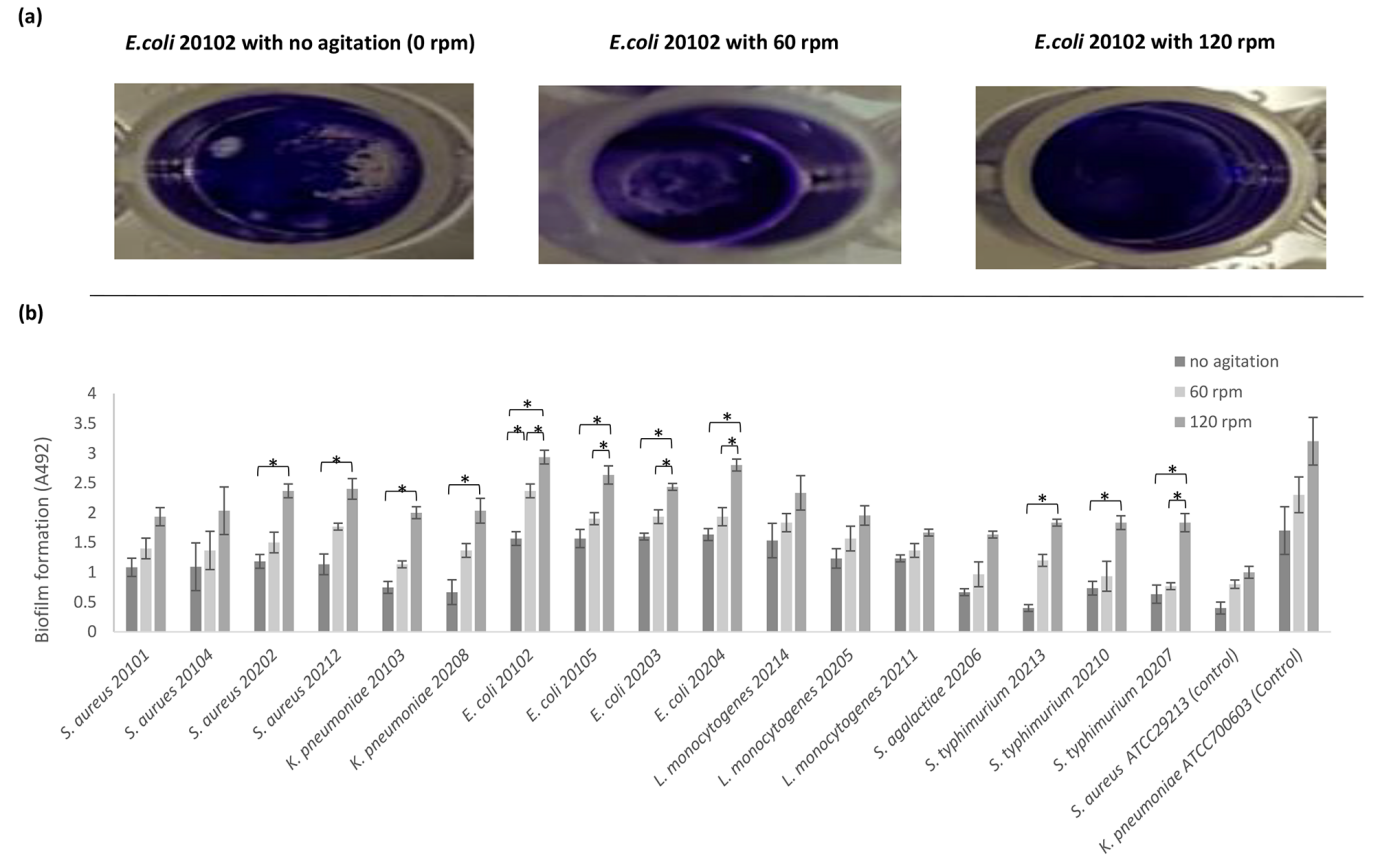
Effect of agitation on biofilm formation of isolated pathogenic bacteria in TSBg. Biofilm formation of E. coli strain 20,102 cultured in TSBg at 30 °C with different levels of agitation (0 rpm, 60 rpm, and 120 rpm) was analyzed in a semi-quantitative manner using crystal violet assays **(a)**. Biofilm production was assessed as the optical density of stained biofilms at 492 nm. Data are presented as mean ± SD of three separate experiments with three replicates per experiment. Asterisks (*) indicate a significant (*p* < 0.01) difference in bacterial biofilm formation **(b)**

Pathogenic bacteria isolated from raw milk demonstrated various degrees of adherence to polystyrene plates consistent with the degree of agitation during incubation (Fig. 2a). All strains grown at 30 °C with 0 rpm, 60 rpm, and 120 rpm agitation exhibited strong biofilm formation. When comparing biofilm formation under 60 rpm agitation to that at 0 rpm, only *E. coli* 20,102 exhibited a significant (*p* < 0.01) increase. However, when comparing the 120 rpm condition to 0 rpm, all isolated pathogenic bacteria exhibited a significant (*p* < 0.01) increase in biofilm formation except for *S. aureus* 20,101, *S. aureus* 20,104, *S. agalactiae* 20,206, and all *L. monocytogenes* strains. When comparing the 120 rpm condition to 60 rpm, all *E. coli* strains and *S. typhimurium* 20,207 exhibited a significant (*p* < 0.01) increase in biofilm formation (Fig. 2b).

## Discussion

Consumers store and transport food practices can affect its safety. Even though experts say to use insulated containers to keep chilled foods cold, especially in warm weather, several consumers don’t do this [28, 29]. Our findings highlight this as a major concern, as pathogen concentrations and biofilm formation were significantly lower when using low-temperature storage (< 10 °C) compared to high-temperature storage (> 10 °C). In addition, we demonstrated that low-temperature storage does not eliminate pathogens, but rather prevents bacterial overgrowth and biofilm formation. These results emphasize the significance of using an ice pack or other cold object to maintain a cool temperature during storage and transportation [28].

Previous studies have shown that many refrigerators are not kept at the recommended temperature, that consumers are not aware of the temperature of their refrigerators, and that milk is often not stored in the coldest part of the refrigerator [30–32]. In this study, we found that more bacteria grew and formed biofilms in milk that was stored at higher temperatures. Other studies have shown that consumers often thaw potentially hazardous foods at room temperature, even though it is recommended to thaw them overnight in the refrigerator [28, 33]. Biddle et al. suggest that the choice of thawing method is important for minimizing bacterial growth in milk [34].

Elmoslemany et al. showed that bacteria that can form biofilms are a major cause of harmful bacteria in dairy production [35]. This means that biofilm-forming bacteria can have a negative impact on the safety and quality of milk and dairy products [36]. Contamination of dairy products is often linked to biofilms forming on the surfaces of milk pipes, cow skin flora, milking containers, and other equipment in dairy manufacturing [37]. Herein, we demonstrated that isolated pathogenic bacteria from raw milk can exhibit increased biofilm formation when the milk is agitated, including by motor vehicle transport. That is, samples transported with more agitation from more distant farms showed more clumped milk and higher bacteria concentrations, which might indicate high biofilm formation.

While our study investigated the impact of temperature and agitation on biofilm formation, a deeper understanding of the underlying mechanisms is crucial. Several studies have shown that environmental factors can influence biofilm formation through gene expression regulation. For instance, milk components have been shown to stimulate biofilm formation in *S. aureus*, with a corresponding upregulation of the *ica* operon (responsible for polysaccharide intercellular adhesin synthesis) under specific conditions [38, 39]. Future research should explore these mechanisms in detail. This could involve identifying and analyzing the expression patterns of genes involved in biofilm formation (e.g., adhesin production, motility, matrix synthesis) under varying temperature and agitation conditions. By elucidating the precise regulatory pathways involved, we can gain a more comprehensive understanding of how environmental factors influence biofilm formation at the molecular level. This knowledge can be vital for developing targeted strategies to prevent or disrupt biofilm formation in clinical and industrial settings.

Several studies have previously isolated *E. coli*, *L. monocytogenes*, *K. pneumoniae*, *S. aureus*, *S. agalactiae*, and *S. typhimurium* from raw milk, consistent with the findings herein [6–8, 19]. However, to the author’s knowledge, this is the first study to examine the effects of temperature and agitation on pathogens in raw milk in the Middle East. A recent study from Australia by Roselyn and coworkers reported that temperature and agitation significantly impact the overgrowth of *E. coli* and *L. monocytogenes* [19]. Our results demonstrate a similar trend for all isolated pathogens. More data are required to substantiate the impact of temperature and agitation on non-pathogenic bacteria such as *Lactococcus* spp. and *Lactobacillus* spp.

Pasteurization, or short-term exposure to high temperature, aims to decrease the number of harmful bacteria to a safe level, giving it a shelf life of about ten days when refrigerated. This heat treatment process guarantees that raw milk is safe microbiologically; however, heat treatment degrades its nutritional value [40]. Several studies have shown that heating milk can change its nutritional value and taste, since several of its molecular components are sensitive to high temperature; in particular, heating milk changes the structure of whey proteins, destroys essential amino acids like lysine, and reduces B vitamins. Therefore, there is interest in developing new strategies that do not rely on heat [15, 40–42]. New methods are being developed to prevent bacteria from growing and forming biofilm on surfaces. These methods include making surfaces smoother to prevent bacterial adhesion, adding antimicrobial coatings, and using anti-adhesive compounds to repel bacteria [15, 42]. In addition, consumer handling practices between sourcing and consumption are a critical element in reducing the risks associated with pathogen contamination and realizing the full benefit of these treatment processes of raw milk [19].Our study demonstrated a high level of agreement (93%) between VITEK-2 and MALDI-TOF MS for identifying isolated pathogens. This concordance rate aligns with previous research, such as the study by Jamal et al. (2014) which reported similar agreement (93.2%) between VITEK-2 and VITEK MS for bacterial identification [43]. However, our findings also highlight the potential for discrepancies. Notably, one isolate (7%) displayed discordant results, with VITEK-2 identifying it as *S. agalactiae* and MALDI-TOF MS identifying it as *Nocardia asteroids*. While this finding aligns with the potential for discrepancies reported by Jamal et al. (2014), it underscores the importance of employing complementary identification methods, especially for critical pathogens like *S. agalactiae* [43]. Further investigation into this specific discrepancy, potentially using gene sequencing, could provide valuable insights into the limitations of each method for specific bacterial strains.

One limitation of this study is that the sample size used (30 mL) may not be representative of the larger volumes that consumers typically handle. Additionally, the small sample size may have affected how quickly the different temperature parameters were reached. Future simulations could be improved by using more representative sample sizes. In this study, we focused on two specific environmental factors, temperature and agitation, however other parameters known to effect bacterial behavior in milk, such as milk pH and vitamin availability, were not measured during this study. These factors are known as potentially impacting both bacterial contamination and biofilm formation. For future studies, we plan to include these additional parameters to increase the analysis and gain more comprehensive understanding.

Our research showed that transporting raw milk at a cold temperature (4 °C) and with less agitation is important for controlling the level of pathogenic bacteria in the milk. Future studies should consider strain variation of pathogenic bacteria and the combined effects of agitation, time, and temperature misuse during the consumer phase on bacterial growth. Public health strategies could be improved by educating consumers about the importance of properly handling raw milk between sourcing and consumption.

## Data Availability

The data underlying this article will be shared on reasonable request to the corresponding author.
